# Investigating the Intersection of Genetic and Social Factors in Cognition Among Mexican Americans

**DOI:** 10.1002/alz70858_104573

**Published:** 2025-12-26

**Authors:** Joey A Contreras, Nancy E Ortega, Humberto Parada, Judy Pa

**Affiliations:** ^1^ Alzheimer's Disease Cooperative Study (ADCS), University of California, San Diego, La Jolla, CA, USA; ^2^ Neurosciences Graduate Program, University of California San Diego, La Jolla, CA, USA; ^3^ San Diego State University, San Diego, CA, USA; ^4^ Alzheimer's Disease Cooperative Study (ADCS), University of California, San Diego, La Jolla, CA, USA

## Abstract

**Background:**

Among Mexican Americans (MA), social determinants of health, such as experiences of discrimination, may contribute to dementia risk above and beyond traditional genetic risk factors like APOE4. For instance, discrimination has been associated with cognitive health outcomes in socio‐economically disadvantaged communities. Due to the increased risk of dementia in Latinos, it is important to investigate how discrimination impacts cognitive health outcomes in this population. We examined the association between perceived everyday discrimination and cognitive function while adjusting for APOE4 among MA and non‐Hispanic white (NHW) adults.

**Method:**

We used baseline data from the community‐based Health and Aging Brain Study‐Health Disparities (HABS‐HD). Cognitive function was assessed using the Spanish‐English Verbal Learning Test (SEVLT), a test of verbal episodic learning and memory. SEVLT scores were converted to z‐scores. Discrimination was assessed using the 9‐item Everyday Discrimination Scale (total scores). We used multivariable linear regression to examine the association between discrimination and cognitive function adjusting for age, sex, and education and APOE4.

**Result:**

We included 221 MA participants [67.9% women, mean age(SD)=61.75(8.24), 24% APOE4 carriers, mean EDS=15.41(6.57)], and 276 NHW participants (59.8% women, mean age(SD)=67.50(8.31), 32% APOE4 carriers, mean EDS=14.65(5.28)). Among all participants, SEVLT scores were not associated with increased perceived discrimination (β=‐0.01, 95% CI[‐0.02, 0.01], *p* = 0.27). By ethnicity, SEVLT scores decreased with increased perceived discrimination among MA participants (β=‐0.02, 95% CI[‐0.04, ‐0.00], *p* = 0.03), but not among NHW participants (β=0.01, 95% CI[‐0.01, ‐0.03], *p* = 0.32).

**Conclusion:**

Perceived discrimination in everyday experiences may be a predictor of cognition among MA, highlighting how social and societal interactions can have a disproportionate impact among certain populations. Future studies should investigate how increased discrimination impacts pathological hallmarks of AD to provide a more mechanistic picture among a population that has a disproportionate burden of dementia.

